# Synthesis of Polyanionic Cellulose Carbamates by Homogeneous Aminolysis in an Ionic Liquid/DMF Medium

**DOI:** 10.3390/molecules27041384

**Published:** 2022-02-18

**Authors:** Cuong Viet Bui, Thomas Rosenau, Hubert Hettegger

**Affiliations:** 1 Institute of Chemistry of Renewable Resources, Department of Chemistry, University of Natural Resources and Life Sciences, Vienna (BOKU), Konrad-Lorenz-Strasse 24, A-3430 Tulln, Austria; cuongvietbui@students.boku.ac.at (C.V.B.); thomas.rosenau@boku.ac.at (T.R.); 2 Department of Food Technology, Faculty of Chemical Engineering, University of Science and Technology, The University of Danang, Danang City 550000, Vietnam; 3 Johan Gadolin Process Chemistry Centre, Åbo Akademi University, Porthansgatan 3, FI-20500 Åbo, Finland

**Keywords:** aminolysis, β-alanine, cellulose carbamate, cellulose carbonate, regioselective synthesis, solid-state NMR, taurine

## Abstract

Polyanionic cellulose carbamates were synthesized by rapid and efficient homogeneous aminolysis of cellulose carbonate half-esters in an ionic liquid/DMF medium. Cellulose *bis*-2,3-*O*-(3,5-dimethylphenyl carbamate), as a model compound, reacted with different chloroformates to cellulose carbonates. These intermediates were subjected to aminolysis, for which both the reactivity of different chloroformates towards C6-OH and the reactivity/suitability of the respective carbonate half-ester in the aminolysis were comprehensively studied. Phenyl chloroformate and 4-chlorophenyl chloroformate readily reacted with C6-OH of the model cellulose derivative, while 4-nitrophenyl chloroformate did not. The intermediate 4-chlorophenyl carbonate derivative with the highest DS (1.05) was then used to evaluate different aminolysis pathways, applying three different amines (propargyl amine, β-alanine, and taurine) as reactants. The latter two zwitterionic compounds are only sparingly soluble in pure DMF as the typical reaction medium for aminolysis; therefore, several alternative procedures were suggested, carefully evaluated, and critically compared. Solubility problems with β-alanine and taurine were overcome by the binary solvent system DMF/[EMIM]OAc (1:1, *v*/*v*), which was shown to be a promising medium for rapid and efficient homogeneous aminolysis and for the preparation of the corresponding cellulose carbamate derivatives or other compounds that are not accessible by conventional isocyanate chemistry. The zwitterionic cellulose carbamate derivatives presented in this work could be promising chiral cation exchangers for HPLC enantiomer separations.

## 1. Introduction

Polysaccharides are produced by many living organisms, including plants, algae, animals, and microorganisms, in which they inter alia serve as an energy/carbon reserve or a structural biopolymer [1]. Within the group of macromolecular carbohydrates, cellulose is the most abundant natural biopolymer with a wide range of applications, rendering it the most important renewable raw material. The major traditional applications of cellulose are paper, board, and fibers. However, the huge potential for novel and advanced applications, especially of chemically modified celluloses, has not yet been fully explored [2].

One type of such high-tech application of cellulose derivatives is in the field of high-performance liquid chromatography (HPLC). Chiral stationary phases (CSPs) based on fully porous or superficially porous inorganic silica particles modified with cellulose derivatives (both by chemical linkage and physical coating) are some of the most powerful and successful separation materials available nowadays [3]. In 1973, the introduction of cellulose acetate as a chiral selector (CS) in chromatography has been a milestone and trailblazer for chiral separations on cellulose derivatives [4]. Since that time, a large number of CSs based on cellulose benzoates and/or phenyl carbamates have been introduced [5,6] and up to 90% of the tested racemic compounds could be successfully separated with cellulose-based and amylose-based CSs [7,8,9]. Regioselectively modified cellulose hetero-substitutes were shown to sometimes even possess higher chiral discrimination than their homo-substituted counterparts [10,11,12,13]. Therefore, several cellulose-hetero-derivatives-based CSs have been developed and used recently, synthesized mostly with two different types of phenyl isocyanates [14,15,16].

**Figure 1:**
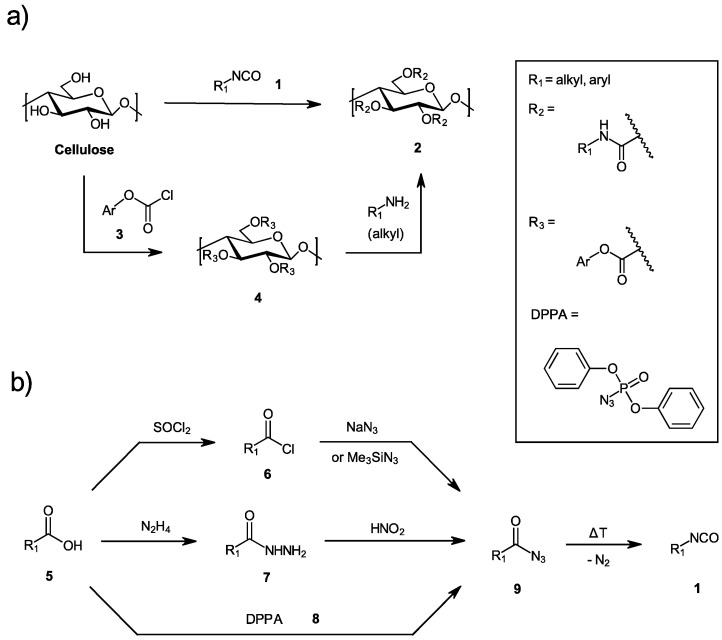
(**a**) Routes towards the synthesis of cellulose carbamates by isocyanate chemistry or carbonate aminolysis; (**b**) Preparation of acyl azides and—after *Curtius* rearrangement—isocyanates from carboxylic acids.

Typically, the (phenyl) carbamates are introduced onto the cellulose backbone by isocyanate chemistry (see Figure 1). However, the accessibility of the carbamate derivatives **2** (also named urethanes or carbanilates) is dependent on the availability of the respective isocyanate compounds **1**.

Commonly used phenyl isocyanates are commercially available (e.g., 3,5-dimethylphenyl isocyanate, 3,5-dichlorophenyl isocyanate, 3-chloro-4-methylphenyl isocyanate, etc.) that render synthesis of CSs based on cellulose phenyl carbamates relatively quick and easy. Another—but a more laborious—approach is the detour via *Curtius*-rearrangement of acyl azides **9**, which can be obtained (1) from the respective aliphatic or aromatic acyl halides **6** by nucleophilic substitution of the halogen for an azide (e.g., with NaN_3_ or azidotrimethylsilane), (2) from acyl hydrazide precursors **7** after reaction with nitrous acid, or (3) by the direct reaction of a carboxylic acid **5** with diphenylphosphoryl azide (DPPA, **8**). A third strategy for the introduction of carbamate groups is the aminolysis of carbonate derivatives **4** (see also Figure 1), which can be obtained by the reaction of cellulose with chloroformates **3**. The aminolysis of cellulose phenyl carbonates as (active) intermediates is an efficient method to introduce carbamate groups onto the cellulose backbone, a reaction that was extensively investigated inter alia by Heinze and others [17,18,19]. One of the most common reaction media for the homogeneous aminolysis of cellulose carbonate derivatives is *N*,*N*-dimethylformamide (DMF), with 24 h of reaction time, due to the good solubility of cellulose carbonate intermediates and reactants in this solvent [18]. However, the use of DMF also limits the choice of reactants (=amine compounds) for a homogeneous aminolysis reaction due to the limited solubility of certain reactants in this dipolar aprotic organic solvent, especially zwitterionic compounds, such as 2-aminopropanoic acid (β-alanine) or 2-aminoethanesulfonic acid (taurine).

Ionic liquids (ILs) have recently emerged as promising media for lignocellulose treatments in biorefinery scenarios and are continuously attracting the enormous interest of the lignocellulose biomass community [20] due to their broad range of applications: fractionation and biorefinery of lignocellulosic biomass [21,22,23,24,25]; cellulose dissolution [26,27,28]; preparation of cellulose fibers, films, beads, and others [20,29,30,31]; and, in particular, the homogeneous synthesis of polysaccharide derivatives [32,33,34,35,36,37]. The latter was employed in esterification reactions [38,39], particularly acetylation [32,40,41,42], but also etherification reactions [43,44,45] and other conversions [20]. The homogeneous syntheses of polysaccharide derivatives facilitated by ionic liquids are fast and with a high degree of efficiency, yielding high degrees of substitution (DS) with short reaction times [20] and good control of the degree of substitution (DS) [46,47]. The homogeneous synthesis of polysaccharide derivatives could be performed in various conventional polysaccharide solvents: *N*,*N*-dimethylacetamide/LiCl, NMMO, dimethyl sulfoxide/tetra-*n*-butylammonium fluoride, and molten inorganic salt hydrates, etc.; nevertheless, ionic liquids have been reported to overcome certain shortcomings of conventional cellulose solvents. Besides the enormous advantages, some drawbacks of especially *N*,*N*-dialkylimidazolium-based ILs were noted, for instance, side reactions with cellulose [20,48], degradation [49], and incompatibility with certain reagents [50].

In the present work, the reaction of cellulose *bis*-2,3-(3,5-dimethylphenyl carbamate) as a cellulose carbamate model derivative and typical intermediate during the preparation of polysaccharide-based carbamate CSs [15,51] with different phenyl chloroformates (4-nitrophenyl chloroformate, 4-chlorophenyl chloroformate, and phenyl chloroformate) was studied. The products, cellulose hetero-derivatives bearing carbonate substituents regioselective at C6, were used as reactive intermediates in aminolysis reactions with zwitterionic compounds (β-alanine and taurine) and propargyl amine (for possible *click* chemical modification). The two amino acids β-alanine and taurine were chosen because their implementation provides weak (carboxylate moiety) and strong (sulfonate functionality) chiral cation exchangers based on polyanionic cellulose carbamates. However, this has not yet been further investigated. Additionally, both reagents are characterized by only limited solubility in the typical aminolysis reaction medium (pure DMF) and, thus, represents a “stumbling block” in contrast to highly reactive and soluble propargylamine. The present work reports how these special amines could be made accessible to carbamate formation despite their special structure and unfavorable solubility characteristics.

## 2. Materials and Methods

### 2.1. Materials

Microcrystalline cellulose (Avicel^®^ PH-101), 1-ethyl-3-methylimidazolium acetate (97%) ([EMIM]OAc), 4-chlorophenyl chloroformate (98%), phenyl chloroformate (97%), 4-nitrophenyl chloroformate (96%), tetra-*n*-butylammonium fluoride hydrate (98%) (TBAF), and propargyl amine (98%) were purchased from Sigma-Aldrich (Schnelldorf, Germany). Microcrystalline cellulose was dried in a vacuum oven at 40 °C for at least two days before use. 3,5-Dimethylphenyl isocyanate (98.5%) and *N*,*O*-*bis*(trimethylsilyl)acetamide (BSA, 98%) were purchased from ABCR GmbH (Karlsruhe, Germany), 3-aminopropionic acid (99%) and 2-aminoethanesulfonic acid (98%) were obtained from TCI Europe N.V. (Zwijndrecht, Belgium). 3-Aminopropionic acid (β-alanine) and 2-aminoethanesulfonic acid (taurine) were ground to a fine powder in a mortar and dried at 40 °C in a vacuum oven for at least two days before use. A regenerated cellulose dialysis membrane (ZelluTrans 6.0, 40-mm flat width, 30-µm wall thickness, nominal MWCO: 6.000–8.000 Da) was purchased from Carl Roth GmbH+ Co. KG (Karlsruhe, Germany). All organic solvents such as *N*,*N*-dimethylacetamide (DMAc), *N*,*N*-dimethylformamide (DMF), and pyridine were reagent grade and dried over 3 Å molecular sieves (Sigma-Aldrich) for at least three days before use. Ethanol (EtOH) and methanol (MeOH) for precipitation and washing were of technical grade and obtained from GmbH + Co. KG or Fisher Scientific (Vienna, Austria).

### 2.2. Instrumentation

ATR-FTIR spectra were recorded on a Frontier IR Single-Range spectrometer (Perkin Elmer, Waltham, MA, USA) equipped with a diamond/ZnSe crystal, LiTaO_3_ detector, and KBr windows. FTIR spectra were evaluated using SpectraGryph software (version v1.2.15). Solid-state ^13^C CP/TOSS NMR experiments were carried out with an Avance III HD instrument (Bruker BioSpin GmbH, Rheinstetten, Germany) with a resonance frequency of 100.68 MHz (^13^C). Data processing was carried out with ACD/NMR Processor Academic Edition 12.01 and TopSpin 3.6.2. Chemical shifts (δ) are given in ppm. Elemental analyses were carried out at the microanalytical laboratory of the University of Vienna on a EURO EA 3000 CHNS-O instrument (HEKAtech, Wegberg, Germany), with halide contents being determined by argentometry.

### 2.3. Synthesis

#### 2.3.1. General Information

Before the addition of reactants, all cellulose derivatives were vigorously stirred in the respective organic solvents under a dry nitrogen atmosphere at RT until a clear solution was formed. All reactions were carried out under an inert atmosphere of dry nitrogen. The precipitation, vacuum filtration, and washing steps during purification of the cellulose derivatives were repeated with the same solvents that were applied for precipitation and obtaining the crude cellulose derivatives. The purified products were dried in a vacuum oven at 40 °C for two days. Any variations or additional steps are described below.

#### 2.3.2. Synthesis of Cellulose *bis*-2,3-*O*-(3,5-Dimethylphenyl Carbamate) **10**

The starting cellulose derivative **10** was synthesized according to Kaida et al. [52], Chassaing et al. [14], and Shen et al. [15]. The respective FTIR and solid-state ^13^C NMR spectra are presented in Figures S1 and S2 (see Supporting Information); the elemental analysis (EA) results, and those of the other cellulose derivatives, are shown in Table 1. All data/spectra are in agreement with the literature data. The DS of 3,5-dimethylphenyl carbamate was calculated to be 1.84 (92%) based on the N content.

#### 2.3.3. Synthesis of Cellulose 6-*O*-(Phenyl Carbonate)-*bis*-2,3-*O*-(3,5-Dimethyl Phenyl Carbamates) **11a–c** (Oxycarbonylation Reaction)

The model cellulose derivative **10** (20.0 g) was dissolved in a mixture of anhydrous DMAc and pyridine (400 mL, 9:1, *v*/*v*) at RT. The solution was cooled to 0 °C by immersion of the round-bottom flask in ice/water. The respective chloroformate derivative (4-nitrophenyl chloroformate (**11a**), 4-chlorophenyl chloroformate (**11b**), or phenyl chloroformate (**11c**), each 2 molar equivalents with respect to the repeating unit of cellulose derivative **10**) was slowly added. The oxycarbonylation reactions were carried out at 0 °C for 12 h. A large excess of either MeOH or EtOH was used to precipitate the crude products, which were then collected by vacuum filtration, washed with either MeOH or EtOH and with distilled water (2× each), and dried in a vacuum oven at 40 °C for two days. Further purification was carried out by redissolution in DMAc, reprecipitation, filtration, and washing.

#### 2.3.4. Synthesis of Cellulose 6-*O*-(Propynyl Carbamate)-*bis*-2,3-*O*-(3,5-Dimethylphenyl Carbamate) **12** (Aminolysis)

Cellulose derivatives **11b** or **11c** (2.5 g) were dissolved in anhydrous DMF (50 mL) at RT. Propargyl amine (5 molar equivalents with respect to the repeating units of cellulose derivatives **11b**/**11c**) was added dropwise at RT. The aminolysis reaction was carried out at 40 °C for 24 h. The crude product was precipitated in a large excess of either MeOH or EtOH, collected by vacuum filtration, washed with either MeOH or EtOH and with distilled water (2× each), and dried at 40 °C in a vacuum oven for two days. Further purification was performed by repeated redissolution (DMF)/reprecipitation, filtration, and washing.

#### 2.3.5. Synthesis of Cellulose Derivatives **13a–b** (Aminolysis)

Procedure A—Heterogeneous Reaction

Cellulose derivative **11b** (2.5 g) was dissolved in anhydrous DMF (40 mL). A solution of propargyl amine in DMF (10 mL, 0.06 molar equivalents with respect to the repeating units of cellulose derivative **11b**) was added dropwise during 10 min and allowed to react for 12 h at 40 °C. Then, crystalline 3-aminopropionic acid (β-alanine, **a**) or 2-aminoethanesulfonic acid (taurine, **b**, each 5 molar equivalents with respect to the repeating units of cellulose derivative **11b**) was added and the heterogeneous mixture was vigorously stirred at 40 °C for 24 h. Crude products were precipitated in distilled water, collected by vacuum filtration, washed with a large excess of distilled water, and subsequently dried at 40 °C in a vacuum oven for two days. Purification of the products (**13aA** and **13bA**) was performed by repeated redissolution/reprecipitation of the crude products in DMF, filtration, and washing.

Procedure B—Homogenous Reaction with *N*,*O*-*bis*(Trimethylsilyl)Acetamide (BSA)

Cellulose derivative **11b** (2.5 g) was dissolved in anhydrous DMF (15 mL). A solution of propargyl amine in DMF (10 mL, 0.06 molar equivalents with respect to the repeating units of cellulose derivative **11b**) was added dropwise during 10 min and allowed to react for 12 h at 40 °C. In parallel, solutions of β-alanine (**a**) or taurine (**b**) were prepared by mixing each compound with DMF (25 mL) (5 molar equivalents with respect to the repeating units of cellulose derivative **11b**). BSA (10 mL in the case of β-alanine or 25 mL in the case of taurine) was added at 80 °C until a clear solution was obtained. Then, the homogeneous reagent solutions were each added to the solution of cellulose derivate **11b** and the homogeneous aminolysis reaction was performed at 40 °C for 24 h. The crude products were precipitated in distilled water, collected by vacuum filtration, washed with a large excess of distilled water, and dried at 40 °C in a vacuum oven for two days. For cleaving off the remaining trimethylsilyl (TMS) groups, the crude products were dissolved in DMF (25 mL), tetra-*n*-butylammonium fluoride (TBAF) was added (5 molar equivalents with respect to the repeating units of the cellulose derivative), and the mixture was vigorously stirred for 24 h at RT. The crude products were precipitated in a large excess of distilled water, collected by vacuum filtration, washed with distilled water, and dried at 40 °C in a vacuum oven for two days. Conversion of salt forms into the corresponding free acids was carried out in a mixture of DMF and 1 M HCl (25 mL, 9:1, *v*/*v*) for 24 h at RT. The products (**13aB** and **13bB**) were isolated by precipitation in distilled water, collected by vacuum filtration, washed with a large excess of distilled water until neutral, and then dried at 40 °C in a vacuum oven for two days.

Procedure C—Two-Step, One-Pot Homogenous Reaction in DMF/[EMIM]OAc

Cellulose derivative **11b** (2.5 g) was dissolved in a mixture of anhydrous DMF (15 mL) and [EMIM]OAc (25 mL). A solution of propargyl amine in DMF (10 mL, 0.06 molar equivalents with respect to the repeating units of cellulose derivative **11b**) was added dropwise during 10 min. DMF acted as one component of the binary reaction medium, additionally reducing the viscosity of [EMIM]OAc. The aminolysis reaction was performed for 2 h at 40 °C. Then, β-alanine or taurine (5 molar equivalents with respect to the repeating units of the cellulose derivative) were added to the solution and the homogeneous aminolysis reaction was continued for 2 h at 40 °C. The crude products were each precipitated in a large excess of distilled water, collected by vacuum filtration, washed with a large excess of distilled water, and then dried at 40 °C in a vacuum oven for two days. The products (**13aC** and **13bC**) were further purified by repeated redissolution/reprecipitation in DMF, filtration, and washing.

Procedure D—Two-Step, One-Pot Homogenous Reaction in DMF and DMF/[EMIM]OAc

Cellulose derivatives **11b** or **11c** (2.5 g) were dissolved in anhydrous DMF (15 mL). A solution of propargyl amine in DMF (10 mL, 0.06 molar equivalents with respect to the repeating units of the cellulose derivatives **11b/11c**) was added dropwise during 10 min. The aminolysis reaction was performed for 12 h at 40 °C. Then, β-alanine or taurine (5 molar equivalents with respect to the repeating units of cellulose derivatives **11b/11c**) were added to the solution and the mixture was vigorously stirred for 15 min. [EMIM]OAc (25 mL) was added and the aminolysis reaction was continued for 2 h at 40 °C. The crude products were each precipitated in a large excess of distilled water, collected by vacuum filtration, washed with a large excess of distilled water, and then dried at 40 °C in a vacuum oven for two days. The crude cellulose derivative **13aD** was purified as described above. Crude cellulose derivative **13bD** was immersed in an aqueous 2% solution of K_2_CO_3_ and shaken at RT for 24 h; then, the crude product was collected by vacuum filtration, washed with a large excess of distilled water until neutral, and then dried in a vacuum oven at 40 °C for two days. The potassium salt derivative was immersed in HCl 0.1 M and allowed to shake at RT for 24 h. Product **13bD** was collected by vacuum filtration, washed with a large excess of distilled water, and then dried at 40 °C in a vacuum oven for two days. For further purification and removal of ionic liquid residues, the crude cellulose derivative **13bD** was dissolved in DMF and dialyzed against water, followed by freeze-drying.

## 3. Results and Discussion

### 3.1. Evaluation of the Oxycarbonylation (Carbonate Formation) Reaction

**Figure 2:**
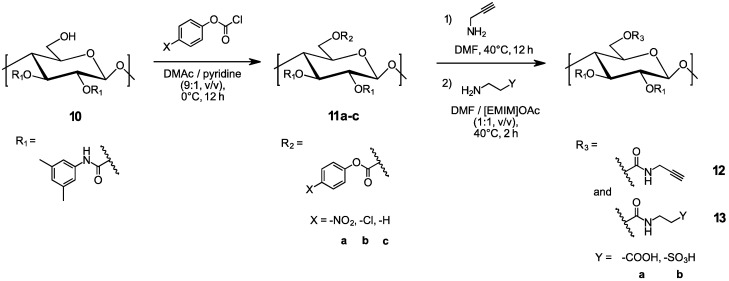
Synthesis of regioselectively substituted, mixed cellulose carbamates by homogeneous aminolysis of the respective carbonate derivatives in DMF or DMF/[EMIM]OAc media.

The reactivity of three phenyl chloroformate reagents (4-nitrophenyl chloroformate, 4-chlorophenyl chloroformate, and phenyl chloroformate, see Figure 2) towards C6-OH of the model cellulose derivative **10** were evaluated in a comparative study as a first step before the actual aminolysis reaction was addressed.

**Figure 3:**
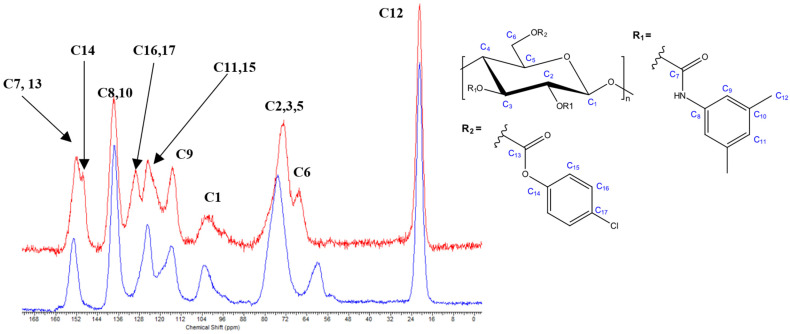
Solid-state ^13^C NMR spectra of cellulose derivative **11b** (red) vs. **10** (blue).

4-Nitrophenyl chloroformate did not exhaustively react with C6-OH of **10**, which was seen by remaining OH group signals at 3529 cm^−1^, C-O groups at 1220 cm^−1^, and the C=O signal at 1723 cm^−1^ in the FTIR spectrum of **11a**. By contrast, 4-chlorophenyl chloroformate and phenyl chloroformate readily reacted to give the intermediate compounds **11b** and **11c**, with the neat conversion being confirmed through the FTIR spectra by the absence of the OH signal at 3529 cm^−1^, the increase in the intensity of the C-O group signal at 1220 cm^−1^ (mixture of carbamate and carbonate), and the signal shift of the C=O group from 1723 cm^−1^ (carbamate) to 1747 cm^−1^ (mixture of carbamate and carbonate) and the presence of the signal at 1488 cm^−1^ assigned to Cl-Ph. In the solid-state ^13^C NMR spectrum of cellulose derivative **11b**, the chemical shift-change of C6 from 60.1 ppm (C6-OH) to the C6-carbonate signal at 67.0 ppm and the additional signals assigned to the *para*-substituted aromatic ring (in particular, C14 at 149.4 ppm, C16 and C17 at 129.5 ppm) indicated a successful carbonate reaction. The FTIR and solid-state ^13^C NMR spectra of cellulose derivatives **11b** vs. **10** are shown in Figure S3 (see Supporting Information) and Figure 3. The DS of 4-chlorophenyl carbonate (based on the Cl content) was calculated to be 1.05. The respective FTIR and solid-state ^13^C NMR spectra of **11c** vs. **10** are presented in Figures S4 and S5, respectively. The DS in the case of the phenyl carbonate (based on EA results, see Supporting Information for the calculation) was 0.97.

In conclusion, both 4-chlorophenyl chloroformate and phenyl chloroformate are suitable reagents for the “activation” of C6-OH in **10** as carbonate, towards subsequent aminolysis; 4-Nitrophenyl chloroformate, on the other hand, was unsuitable.

### 3.2. Aminolysis Reaction with Propargyl Amine

**Figure 4:**
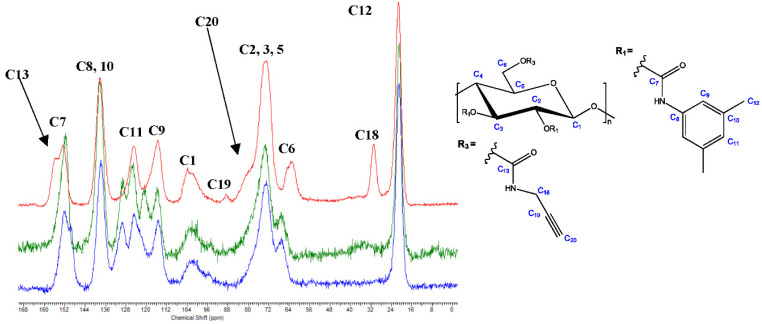
Solid-state ^13^C NMR spectra of cellulose derivative **12** (red) vs. **11c** (green) and **11b** (blue).

The aminolysis reaction between propargyl amine and cellulose derivatives **11b** and **11c** was performed under homogeneous reaction conditions, owing to the good solubility of the reaction partners in DMF. This reaction served as a reference for procedures **A**–**D** (see experimental section), both regarding successful conversion and spectral information. The FTIR and solid-state ^13^C NMR spectra of cellulose derivatives **12** vs. **11b** and **11c** are presented in Figure S6 and Figure 4, respectively. Successful aminolysis was inter alia indicated by the change of the C=O IR band from 1748 cm^−1^ (superposition of carbamate and carbonate) to 1713 cm^−1^ (carbamate only), and the absence of the Cl-Ph signal at 1488 cm^−1^.

Furthermore, in the solid-state ^13^C NMR spectrum of cellulose derivative **12**, additional signals for C13 (155.3 ppm), C18 (30.8 ppm), C19 (81.0 ppm), and C20 (88.7 ppm) occurred while the signals of the aromatic ring of the carbonate derivatives were absent (see above for cellulose derivatives **11b** and **11c**). The chemical shift of C6 changed from approx. 67.0 ppm (carbonate) to 62.8 ppm (carbamate), which also proved that the aminolysis reaction was successful.

### 3.3. Aminolysis Procedures A–D

Cellulose derivative **11b** with the highest carbonate DS (1.05) was used to evaluate different aminolysis procedures to synthesize cellulose derivatives **13a** (with β-alanine) and **13b** (taurine). Both reagents had limited solubility in the “typical” aminolysis reaction medium DMF and represented a “stumbling block” in contrast to highly reactive and soluble propargylamine.

Procedure A—Heterogeneous Comparison

With β-alanine and taurine being insoluble in the reaction medium DMF, the aminolysis reaction was attempted under heterogeneous conditions as a control experiment. After purification, the obtained products were similar to the starting materials according to their spectra; thus, it was obvious that the heterogeneous aminolysis had not been successful because of the limited solubility of the zwitterionic compounds. Thus, derivatization by trimethylsilylation with BSA, described inter alia by Hoffmann et al. [53], was employed to overcome these solubility issues.

Procedure B—Silylation Followed by Aminolysis

The solubility issues of β-alanine and taurine in DMF were resolved by the silylation of the amino acid derivatives with BSA so that aminolysis could be carried out under homogeneous reaction conditions. The FTIR and solid-state ^13^C NMR spectra of products **13aB** (aminolysis with β-alanine) vs. **11b** (carbonate-type starting material) are shown in Figures S7 and S8. The IR vibration shift of the C=O stretch from 1748 cm^−1^ (superposition of carbamate and carbonate) to 1721 cm^−1^ (carbamate only), the relative decrease in C-O intensity due to cleavage of the 4-chlorophenyl carbonate moiety, and the absence of the Cl-Ph signal at 1488 cm^−1^ indicated that the homogeneous aminolysis reaction with TMS-derivatized β-alanine was—at least partially—successful. The solid-state ^13^C NMR spectra indicated the presence of remaining trimethylsilyl (TMS) groups (~ 0 ppm) in the crude product and tetra-*n*-butylammonium (TBA) groups at 13.5 and 59.6 ppm after desilylation with TBAF, which was attributed to TBA counterions before protonation upon the acidic workup. The three carbon signals of the amino acid moiety (36.0, 39.7, and 174.7 ppm) in the product were visible. The DS of 3-propionic acid carbamate based on the N content was calculated to be 0.43.

While the homogeneous aminolysis reaction was successful with trimethylsilylated β-alanine, it failed in the case of taurine. The FTIR spectrum indicated that the 4-chlorophenyl carbonate moiety was cleaved off (1488 cm^−1^) but the S content of the hypothetical product **13bB** was very low (0.08 ± 0.04%).

Procedure C—Two-Step, One-Pot Homogenous Reaction in DMF/[EMIM]OAc

Due to the above solubility issues, the use of an ionic liquid (IL) as a solvent or component of a solvent mixture seemed promising. Several ILs were tested and [EMIM]OAc was finally chosen for the homogeneous aminolysis reaction due to the good solubility of both cellulose derivative **11b** and the amine compounds (β-alanine and taurine) in an [EMIM]OAc/DMF mixture. Commercial availability was another advantage in this regard.

The FTIR and solid-state ^13^C NMR spectra of cellulose derivatives **13aC** and **13bC** vs. **11b** are presented in Figures S11–S14. In general, the aminolysis reaction with propargyl amine and β-alanine/taurine proceeded satisfactorily in a mixture of DMF and [EMIM]OAc (1:1, *v*/*v*). Both analytical techniques confirmed a successful aminolysis reaction and substitution, as seen by the C=O band shifting from 1748 cm^−1^ (superposition of carbamate and carbonate) to 1728 cm^−1^ (carbamate only), the overall increase of the C=O signal (carbamate and COOH of the β-alanine moiety), and the absence of the signal at 1488 cm^−1^ (Cl-Ph) as well as the appearance of a band at 1035 cm^−1^ assigned to the S=O group in case of taurine. New ^13^C NMR resonances in the solid-state spectra of **13aC** and **13bC** were assigned to C18 at ca. 30–31 ppm, C21 and C22 at approx. 36 ppm, and C23 at 173.8 ppm, as well as C24 at 36.8 and C25 at 50.9 ppm in the case of taurine. An additional signal at 55.2 ppm in the solid-state ^13^C NMR spectra of derivative **13aC** (and also in case of **13bC**, see Figure S14), with a shift typical of OCH_3_ groups, cannot be explained so far. The signal persisted after extensive purification by repetitive redissolution and protonation in DMF and 1 M HCl (9:1, *v*/*v*) followed by precipitation and washing with distilled water. This phenomenon was only observed when [EMIM]OAc was added for promoting the aminolysis reaction and not in pure DMF, as was used in the case of propargylamine and procedures **A** and **B**. Liquid-state 2D NMR studies (HMBC) were not conclusive either (note: the cellulose derivatives **13aB**, **13aC**, and **13bC** were soluble in tetrahydrofuran but not in EtOH or MeOH). This signal is attributed to a by-product from side reactions between cellulose derivative **11b** and [EMIM]OAc; further studies to elucidate its origin are ongoing.

The estimated DS of the 3-propionic acid carbamate calculated by the N content was 0.62, and 0.29 in the case of the 2-ethanesulfonic acid carbamate (based on the S content). These results show that the reaction, as such, was successful; however, a quantitative conversion/substitution was not possible by this two-step, one-pot homogenous reaction in DMF/[EMIM]OAc.

Procedure D—Two-Step, One-Pot Homogenous Reaction in DMF and DMF/[EMIM]OAc

**Figure 5:**
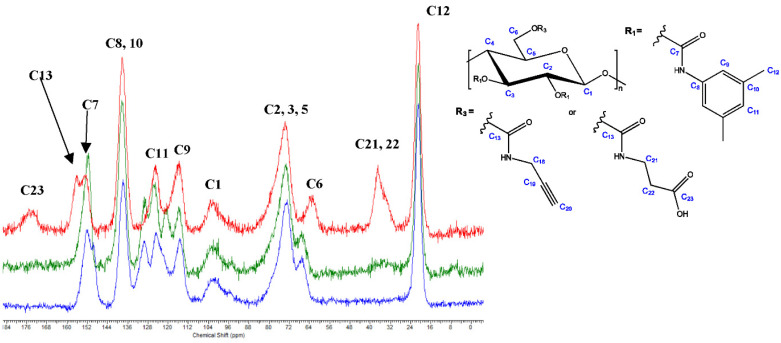
Solid-state ^13^C NMR spectra of cellulose derivatives **13aD** (red) vs. **11c** (green) and **11b** (blue).

To increase the DS of the amino acid carbamates, the aminolysis reactions of propargyl amine and β-alanine/taurine were each carried out consecutively in different reaction media: first, with propargyl amine in DMF; then, with the zwitterionic amino acids in a mixture of DMF and [EMIM]OAc). The FTIR and solid-state ^13^C NMR spectra of the corresponding cellulose derivative **13aD** (β-alanine case) vs. **11b** and **11c** are presented in Figure S15 and Figure 5 . Both techniques confirmed that the aminolysis reactions were successful when carried out according to a two-step, one-pot method with the addition of [EMIM]OAc after the first step.

Resonances (^13^C) assigned to C23 at 175.1, C13 at 155.9, C21 and C22 at approx. 36 ppm, and the chemical shift change of C6 from approx. 67 ppm (carbonate) to 62.5 ppm (carbamate), as well as the absence of the respective 4-chlorophenyl and phenyl carbonate signals in the aromatic region, indicated a neat process. The DS of 3-propionic acid carbamate calculated by the N content was 0.94—an almost quantitative carbonate-to-carbamate conversion.

**Figure 6:**
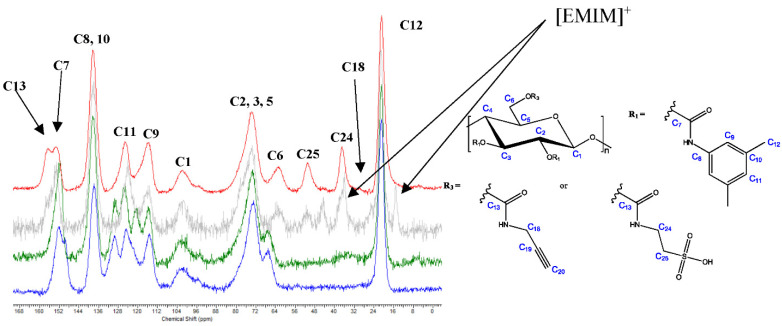
Solid-state ^13^C NMR spectra of cellulose derivatives **13bD** (grey = crude product, red = after purification) vs. **11c** (green) and **11b** (blue).

Moreover, the homogeneous aminolysis reaction with propargyl amine followed by taurine in the above-described, two-step manner with a change in the solvent composition was successful. The respective FTIR and solid-state ^13^C NMR spectra of cellulose derivatives **13bD** vs. **11b** and **11c** are shown in Figure S16 and Figure 6 . ^13^C resonances assigned to C13 at 156.7 (C6-carbamate), C18 at 30.3, C24 at 37.0, and C25 at 51.0 ppm were proof of the successful aminolysis reaction. The solid-state ^13^C NMR spectrum of the crude derivative **13bD** after precipitation still showed signals assigned to [EMIM]^+^ at 44.5 and 15.1 ppm; thus, an additional purification/protonation step was necessary to obtain the neat polyanionic compound **13bD**. As the target compound could neither be precipitated in distilled water nor in EtOH or MeOH after redissolution in a mixture of DMF and 1 M HCl (9:1, *v*/*v*), it was treated with aqueous 2% K_2_CO_3_ to extract residual phenolic compounds and exchange the cations. Subsequent protonation in 0.1 M HCl followed by dialysis through a cellulose membrane to extract small by-products and fragments yielded the pure target compound. The DS of 2-ethanesulfonic acid carbamate based on the S content was 0.92, which is close to quantitative conversion as well. Note that cellulose derivative **13aD** (β-alanine-type) was soluble in tetrahydrofuran, EtOH, and MeOH, while cellulose derivative **13bD** (taurine-type) was soluble in EtOH and MeOH but insoluble in tetrahydrofuran. The newly used binary DMF/ionic liquid medium thus allowed conducting the reaction homogeneously and further expand the possibilities of the aminolysis reaction for the preparation of cellulose carbamates.

## 4. Conclusions and Outlook

In this work, ATR-FTIR and solid-state ^13^C CP/TOSS NMR have been used to evaluate the synthesis of cellulose carbonate half-esters and their subsequent aminolysis. The chemical structure of the intermediates and the target products—polyanionic cellulose carbamates—was confirmed. The carbonate half-ester-formation between C6-OH of the starting cellulose derivative *bis*-2,3-*O*-(3,5-dimethylphenyl carbamate) with different chloroformate reagents (4-nitrophenyl, 4-chlorophenyl, and phenyl chloroformate) was evaluated. Phenyl chloroformate and 4-chlorophenyl chloroformate were shown to react readily, while 4-nitrophenyl chloroformate did not. Different chemical synthesis strategies for a subsequent aminolysis reaction of the cellulose carbonate half-ester derivatives using propargyl amine (as a chemical anchor for *click* synthons), β-alanine (as a weak ion exchanger), and taurine (as a strong ion exchanger) have been studied. The two-step, one-pot approach in DMF and [EMIM]OAc/DMF was shown to be the most suitable protocol for the homogeneous aminolysis reactions, being rapid and efficient, as β-alanine and taurine are well-soluble in a [EMIM]OAc/DMF mixture but only sparingly in DMF. The degrees of substitution of the respective alanine and taurine moieties in the polyanionic cellulose carbamates were 0.94 (3-propionic acid carbamate) and 0.92 (2-ethanesulfonic acid carbamate), respectively. With these optimized protocols in hand, it is now possible to further expand the possibilities for functional carbamate groups in cellulose derivatives for use as chiral selectors in HPLC enantiomer separation applications, beyond the standard functionalities accessible through isocyanate reagents.

## Figures and Tables

**Table 1:** Results of elemental analysis.

	Calculated (wt%)						Found (wt%)					
	C	H	O	N	Cl	S	C	H	O	N	Cl	S
**10**	63.15	6.18	24.53	6.14	-	-	59.90 ± 0.07	6.27 ± 0.05	25.96 ± 0.50	5.66 ± 0.07	-	-
**11b**	60.93	5.11	23.57	4.58	5.80	-	59.43 ± 0.14	4.86 ± 0.18	25.96 ± 0.50	4.28 ± 0.12	6.11 ± 0.05	-
**11c**	64.57	5.59	24.97	4.86	-	-	61.27 ± 0.10	5.08 ± 0.14	25.16 ± 0.34	4.48 ± 0.04	-	-
**13aB**	58.84	5.82	27.99	7.35	-	-	57.79 ± 0.05	5.98 ± 0.09	28.34 ± 0.09	5.56 ± 0.03	-	-
**13aC**							59.96 ± 0.18	5.98 ± 0.12	26.39 ± 0.50	6.03 ± 0.05	-	-
**13aD**							55.04 ± 0.19	5.56 ± 0.12	29.48 ± 0.64	6.81 ± 0.06	-	-
**13bB**	53.37	5.47	28.96	6.92	-	5.28	59.89 ± 0.14	6.13 ± 0.03	26.01 ± 0.72	6.11 ± 0.02	-	0.08 ± 0.04
**13bC**							49.75 ± 0.19	5.15 ± 0.03	23.48 ± 0.52	5.41 ± 0.03	-	1.03 ± 0.02
**13bD**							46.58 ± 0.02	5.48 ± 0.08	32.70 ± 1.17	6.27 ± 0.03	-	4.86 ± 0.04

Note: Deviations from the calculated values are the result of incomplete substitution.

## Data Availability

Not applicable.

## Supplementary Materials

The following supporting information can be downloaded online: Figure S1. FTIR spectrum of cellulose derivative **10**; Figure S2. Solid-state ^13^C NMR spectrum of cellulose derivative **10**; Figure S3. FTIR spectra of cellulose derivatives **11b** vs. **10**; Figure S4. FTIR spectra of cellulose derivatives **11c** vs. **10**; Figure S5. Solid-state ^13^C NMR spectra of cellulose derivatives **11c** vs. **10**; Figure S6. FTIR spectrum of cellulose derivatives **12** vs. **11b** and cellulose derivative **11c**; Figure S7. FTIR spectra of cellulose derivatives **13aB** vs. **11b**; Figure S8. Solid-state ^13^C NMR spectra of cellulose derivatives **13aB** vs. **11b**; Figure S9. FTIR spectra of cellulose derivatives **13bB** vs. **11b**; Figure S10. Solid-state ^13^C NMR spectra of cellulose derivatives **13bB** vs. **11b**; Figure S11. FTIR spectra of cellulose derivatives **13aC** vs. **11b**; Figure S12. Solid-state ^13^C NMR spectra of cellulose derivative **13aC** vs. **11b**; Figure S13. FTIR spectra of cellulose derivatives **13bC** vs. **11b**; Figure S14. Solid-state ^13^C NMR spectra of cellulose derivatives **13bC** vs. **11b**; Figure S15. FTIR spectra of cellulose derivative **13aD** vs. **11b** and **11c**; Figure S16. FTIR spectra of cellulose derivative **13bD** vs. **11b** and **11c**.

## Abbreviations

BSA	*N*,*O*-*Bis*-(trimethylsilyl)acetamide
CS	Chiral selector
DMAc	*N*,*N*-Dimethylacetamide
DMF	*N*,*N*-Dimethylformamide
DS	Degree of substitution
EA	Elemental analysis
[EMIM]OAc	1-Ethyl-3-methylimidazolium acetate
EtOH	Ethanol
MeOH	Methanol
IL	Ionic liquid
RT	Room temperature
TBAF	tetra-*n*-Butylammonium fluoride hydrate

## References

[1] Heinze T., Liebert T., Koschella A. (2006). Esterification of Polysaccharides.

[2] Elschner T., Heinze T. (2015). Cellulose carbonates: A platform for promising biopolymer derivatives with multifunctional capabilities. Macromol. Biosci..

[3] Hettegger H., Lindner W., Rosenau T., Rauter A.P., Christensen B., Somsak L., Kosma P., Adamo R. (2020). Derivatized polysaccharides on silica and hybridized with silica in chromatography and separation—A mini review. Recent Trends in Carbohydrate Chemistry: Synthesis, Structure and Function of Carbohydrates.

[4] Lüttringhaus A., Hess U., Rosenbaum H.J.I. (1967). Mitt.: Optisch aktives 4.5. 6.7-Dibenzo-1.2-dithiacyclooctadien. Z. Für. Nat. B.

[5] Fanali C., D’Orazio G., Gentili A., Fanali S. (2019). Analysis of Enantiomers in Products of Food Interest. Molecules.

[6] Minguillón C., Franco P., Oliveros L., López P. (1996). Bonded cellulose-derived high-performance liquid chromatography chiral stationary phases I. Influence of the degree of fixation on selectivity. J. Chromatogr. A.

[7] Yin C., Chen W., Zhang J., Zhang M., Zhang J. (2019). A facile and efficient method to fabricate high-resolution immobilized cellulose-based chiral stationary phases via thiol-ene click chemistry. Sep. Purif. Technol..

[8] Ikai T., Okamoto Y. (2009). Structure control of polysaccharide derivatives for efficient separation of enantiomers by chromatography. Chem. Rev..

[9] Shen J., Okamoto Y. (2016). Efficient separation of enantiomers using stereoregular chiral polymers. Chem. Rev..

[10] Yin C., Zhang J., Chang L., Zhang M., Yang T., Zhang X., Zhang J. (2019). Regioselectively substituted cellulose mixed esters synthesized by two-steps route to understand chiral recognition mechanism and fabricate high-performance chiral stationary phases. Anal. Chim. Acta.

[11] Felix G. (2001). Regioselectively modified polysaccharide derivatives as chiral stationary phases in high-performance liquid chromatography. J. Chromatogr. A.

[12] Acemoglu M., Küsters E., Baumann J., Hernandez I., Pong Mak C. (1998). Synthesis of regioselectively substituted cellulose derivatives and applications in chiral chromatography. Chirality.

[13] Katoh Y., Tsujimoto Y., Yamamoto C., Ikai T., Kamigaito M., Okamoto Y. (2011). Chiral recognition ability of cellulose derivatives bearing pyridyl and bipyridyl residues as chiral stationary phases for high-performance liquid chromatography. Polym. J..

[14] Chassaing C., Thienpont A., Félix G. (1996). Regioselective carbamoylated and benzoylated cellulose for the separation of enantiomers in high-performance liquid chromatography. J. Chromatogr. A.

[15] Shen J., Wang F., Bi W., Liu B., Liu S., Okamoto Y. (2018). Synthesis of cellulose carbamates bearing regioselective substituents at 2, 3-and 6-positions for efficient chromatographic enantioseparation. J. Chromatogr. A.

[16] Zheng J., Bragg W., Hou J., Lin N., Chandrasekaran S., Shamsi S.A. (2009). Sulfated and sulfonated polysaccharide as chiral stationary phases for capillary electrochromatography and capillary electrochromatography–mass spectrometry. J. Chromatogr. A.

[17] Ganske K., Heinze T. (2018). Evaluation of the Synthesis of Soluble Aromatic Cellulose Carbonates of Low Degree of Substitution. Macromol. Chem. Phys..

[18] Elschner T., Ganske K., Heinze T. (2013). Synthesis and aminolysis of polysaccharide carbonates. Cellulose.

[19] Pourjavadi A., Seidi F., Afjeh S.S., Nikoseresht N., Salimi H., Nemati N. (2011). Synthesis of soluble N-functionalized polysaccharide derivatives using phenyl carbonate precursor and their application as catalysts. Starch-Stärke.

[20] Gericke M., Fardim P., Heinze T. (2012). Ionic liquids—promising but challenging solvents for homogeneous derivatization of cellulose. Molecules.

[21] Schrems M., Brandt A., Welton T., Liebner F., Rosenau T., Potthast A. (2011). Ionic liquids as media for biomass processing: Opportunities and restrictions. Holzforschung.

[22] Brandt A., Erickson J.K., Hallett J.P., Murphy R.J., Potthast A., Ray M.J., Rosenau T., Schrems M., Welton T. (2012). Soaking of pine wood chips with ionic liquids for reduced energy input during grinding. Green Chem..

[23] Stark A. (2011). Ionic liquids in the biorefinery: A critical assessment of their potential. Energy Environ. Sci..

[24] Mora-Pale M., Meli L., Doherty T.V., Linhardt R.J., Dordick J.S. (2011). Room temperature ionic liquids as emerging solvents for the pretreatment of lignocellulosic biomass. Biotechnol. Bioeng..

[25] Lopes J.M., Bermejo M.D., Martín Á., Cocero M.J. (2017). Ionic liquid as reaction media for the production of cellulose-derived polymers from cellulosic biomass. ChemEngineering.

[26] Koide M., Wataoka I., Urakawa H., Kajiwara K., Henniges U., Rosenau T. (2019). Intrinsic characteristics of cellulose dissolved in an ionic liquid: The shape of a single cellulose molecule in solution. Cellulose.

[27] Kosan B., Michels C., Meister F. (2008). Dissolution and forming of cellulose with ionic liquids. Cellulose.

[28] Fukaya Y., Hayashi K., Wada M., Ohno H. (2008). Cellulose dissolution with polar ionic liquids under mild conditions: Required factors for anions. Green Chem..

[29] Pang J.-H., Liu X., Wu M., Wu Y.-Y., Zhang X.-M., Sun R.-C. (2014). Fabrication and characterization of regenerated cellulose films using different ionic liquids. J. Spectrosc..

[30] Cao Y., Li H., Zhang Y., Zhang J., He J. (2010). Structure and properties of novel regenerated cellulose films prepared from cornhusk cellulose in room temperature ionic liquids. J. Appl. Polym. Sci..

[31] Gericke M., Trygg J., Fardim P. (2013). Functional cellulose beads: Preparation, characterization, and applications. Chem. Rev..

[32] Stepan A.M., King A.W., Kakko T., Toriz G., Kilpeläinen I., Gatenholm P. (2013). Fast and highly efficient acetylation of xylans in ionic liquid systems. Cellulose.

[33] Zhang J., Wu J., Cao Y., Sang S., Zhang J., He J. (2009). Synthesis of cellulose benzoates under homogeneous conditions in an ionic liquid. Cellulose.

[34] Peng X., Ren J., Zhong L., Sun R. (2011). Homogeneous synthesis of hemicellulosic succinates with high degree of substitution in ionic liquid. Carbohydr. Polym..

[35] Cao Y., Li H., Zhang J. (2011). Homogeneous synthesis and characterization of cellulose acetate butyrate (CAB) in 1-allyl-3-methylimidazolium chloride (AmimCl) ionic liquid. Ind. Eng. Chem. Res..

[36] Singh R.K., Gupta P., Sharma O.P., Ray S.S. (2015). Homogeneous synthesis of cellulose fatty esters in ionic liquid (1-butyl-3-methylimidazolium chloride) and study of their comparative antifriction property. J. Ind. Eng. Chem..

[37] Peng X., Ren J., Sun R. (2010). Homogeneous esterification of xylan-rich hemicelluloses with maleic anhydride in ionic liquid. Biomacromolecules.

[38] Zhang J., Chen W., Feng Y., Wu J., Yu J., He J., Zhang J. (2015). Homogeneous esterification of cellulose in room temperature ionic liquids. Polym. Int..

[39] Lacroix C., Sultan E., Fleury E., Charlot A. (2012). Functional galactomannan platform from convenient esterification in imidazolium-based ionic liquids. Polym. Chem..

[40] Zweckmair T., Hettegger H., Abushammala H., Bacher M., Potthast A., Laborie M.-P., Rosenau T. (2015). On the mechanism of the unwanted acetylation of polysaccharides by 1, 3-dialkylimidazolium acetate ionic liquids: Part 1—Analysis, acetylating agent, influence of water, and mechanistic considerations. Cellulose.

[41] Mine S., Izawa H., Kaneko Y., Kadokawa J. (2009). Acetylation of α-chitin in ionic liquids. Carbohydr. Res..

[42] Ren J., Sun R., Liu C., Cao Z., Luo W. (2007). Acetylation of wheat straw hemicelluloses in ionic liquid using iodine as a catalyst. Carbohydr. Polym..

[43] Mormann W., Wezstein M. (2009). Trimethylsilylation of cellulose in ionic liquids. Macromol. Biosci..

[44] Gömez J.A.C., Erler U.W., Klemm D.O. (1996). Physics. 4-methoxy substituted trityl groups in 6-O protection of cellulose: Homogeneous synthesis, characterization, detritylation. Macromol. Chem..

[45] Gericke M., Liebert T., Heinze T. (2009). Interaction of ionic liquids with polysaccharides, 8–synthesis of cellulose sulfates suitable for polyelectrolyte complex formation. Macromol. Biosci..

[46] Liebert T.F., Heinze T.J. (2001). Exploitation of reactivity and selectivity in cellulose functionalization using unconventional media for the design of products showing new superstructures. Biomacromolecules.

[47] Liebert T.F., Heinze T. (2005). Tailored cellulose esters: Synthesis and structure determination. Biomacromolecules.

[48] Ebner G., Schiehser S., Potthast A., Rosenau T. (2008). Side reaction of cellulose with common 1-alkyl-3-methylimidazolium-based ionic liquids. Tetrahedron Lett..

[49] Liebner F., Patel I., Ebner G., Becker E., Horix M., Potthast A., Rosenau T. (2010). Thermal aging of 1-alkyl-3-methylimidazolium ionic liquids and its effect on dissolved cellulose. Holzforschung.

[50] Böhmdorfer S., Hosoya T., Röder T., Potthast A., Rosenau T. (2017). A cautionary note on thermal runaway reactions in mixtures of 1-alkyl-3-methylimidazolium ionic liquids and N-methylmorpholine-N-oxide. Cellulose.

[51] Dai X., Bi W., Sun M., Wang F., Shen J., Okamoto Y. (2019). Chiral recognition ability of amylose derivatives bearing regioselectively different carbamate pendants at 2, 3-and 6-positions. Carbohydr. Polym..

[52] Kaida Y., Okamoto Y. (1993). Optical resolution on regioselectively carbamoylated cellulose and amylose with 3, 5-dimethylphenyl and 3, 5-dichlorophenyl isocyanates. Bull. Chem. Soc. Jpn..

[53] Hoffmann C.V., Pell R., Lämmerhofer M., Lindner W. (2008). Synergistic effects on enantioselectivity of zwitterionic chiral stationary phases for separations of chiral acids, bases, and amino acids by HPLC. Anal. Chem..

