# Supplementary material for: Synthesis of Polyanionic Cellulose Carbamates by Homogeneous Aminolysis in an Ionic Liquid/DMF Medium
Source: Molecules. 2022 Feb 18;27(4):1384. doi: 10.3390/molecules27041384 (PMC8876763; doi:10.3390/molecules27041384)
Supplement: Supplementary file 1 [file molecules-27-01384-s001.zip › molecules-1520101-supplementary.pdf]

Supporting Information

Synthesis of Polyanionic Cellulose Carbamates by Homogeneous Aminolysis in an Ionic Liquid/DMF Medium

Cuong Viet Bui[1,2], Thomas Rosenau[1,3], Hubert Hettegger[1,*]

[1] Institute of Chemistry of Renewable Resources, Department of Chemistry, University of Natural Resources and Life Sciences, Vienna (BOKU), Konrad-Lorenz-Strasse 24, A-3430 Tulln, Austria

[2] Department of Food Technology, Faculty of Chemical Engineering, University of Science and Technology, The University of Danang, Danang City, 550000, Vietnam

[3] Johan Gadolin Process Chemistry Centre, Åbo Akademi University, Porthansgatan 3, FI-20500 Åbo, Finland

[*] Correspondence: hubert.hettegger@boku.ac.at

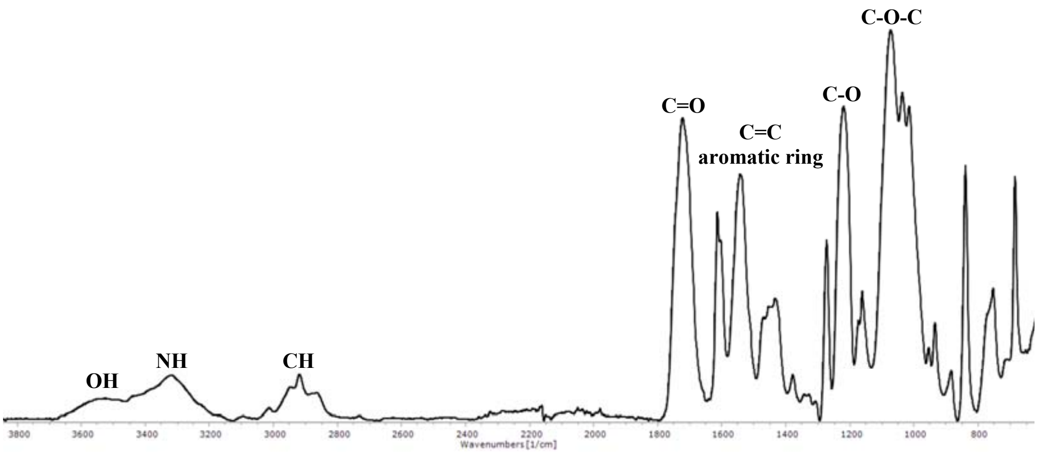

Fig. S1. FTIR spectrum of cellulose derivative **10**,

υ = 3529 (OH), 3318 (NH), 2919 (CH), 1723 (C=O), 1614, 1543, 1434,

839 (C=C aromatic ring), 1220 (C-O), and 1073 cm^{-1} (C-O-C).

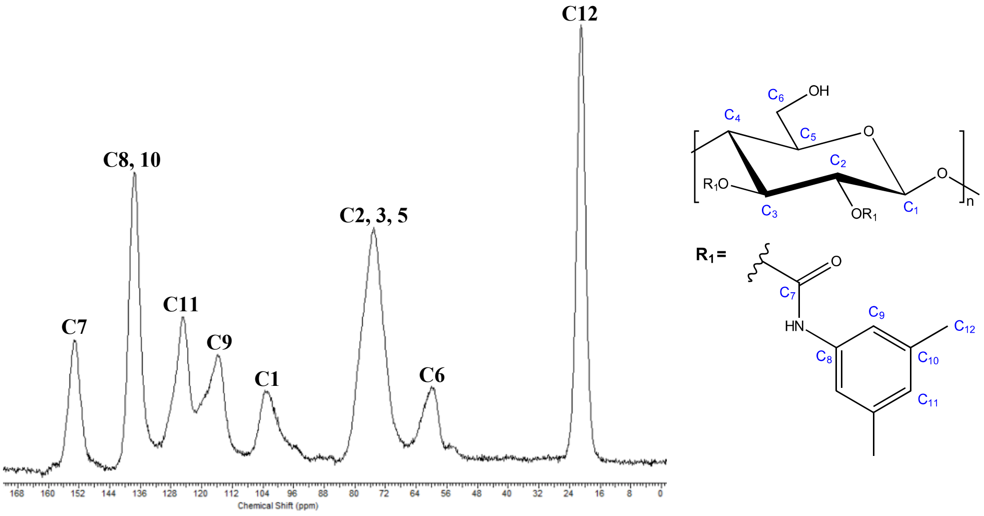

Fig. S2. Solid-state ^{13}C NMR spectrum of cellulose derivative **10**,

δ = 103.5 (C1), 75.1 (C2, 3, 5), 60.1 (C6), 153.3 (C7), 137.7 (C8, 10), 115.8 (C9),

125.0 (C11), and 21.0 ppm (C12).

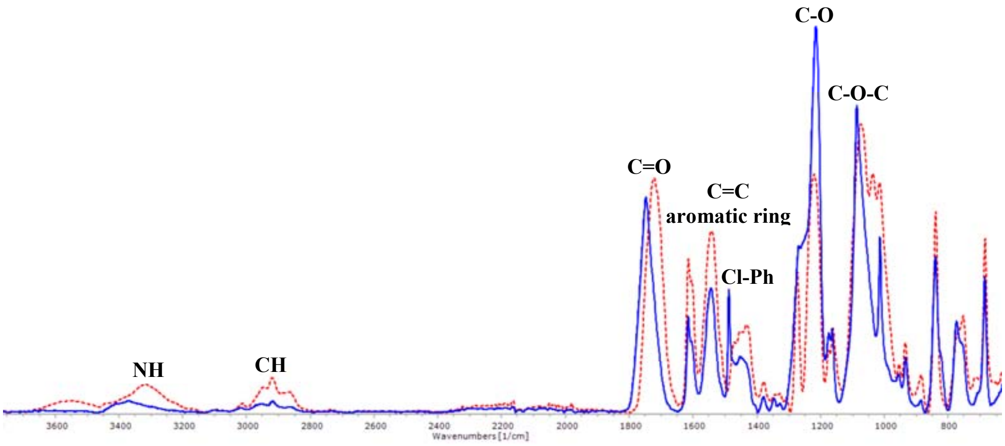

Fig. S3. FTIR spectra of cellulose derivatives **11b** (blue) *vs.* **10** (dashed red),

Cellulose derivative **11b**: υ = 3369 (NH), 2918 (CH), 1748 (C=O), 1615, 1543, 1447, 840 (C=C aromatic ring), 1488 (Cl-Ph), 1215 (C-O), and 1087 cm^{-1} (C-O-C).

Solid-state ^{13}C NMR of cellulose derivative **11b**: δ = 101.5 (C1), 72.9 (C2, 3, 5), 67.0 (C6), 151.7 (C7, 13), 137.9 (C8, 10), 115.5 (C9), 124.9 (C11, 15), 21.0 (C12), 149.4 (C14), and 129.5 ppm (C16, 17).

Obtained: 26.06 g, yield: 97.3 %.

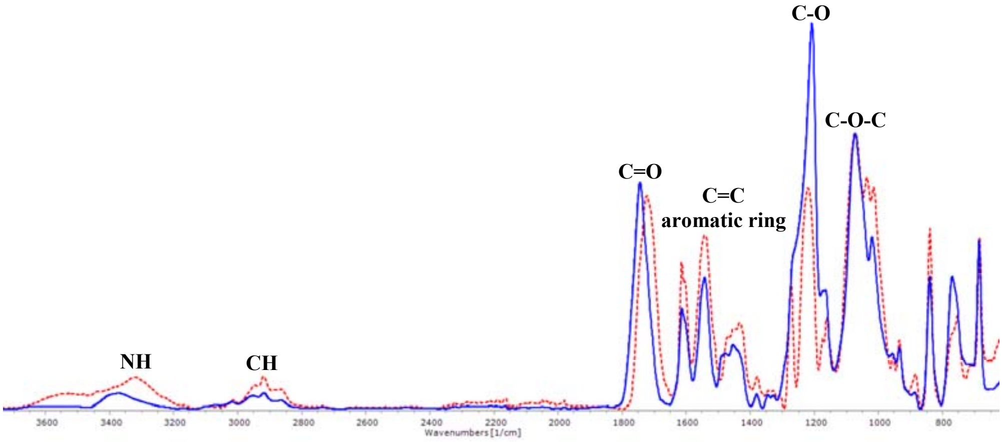

Fig. S4. FTIR spectra of cellulose derivatives **11c** (blue) *vs.* **10** (dashed red),

Cellulose derivative **11c**: υ = 3369 (NH), 2918 (CH), 1747 (C=O), 1613, 1543, 1454, 840 (C=C aromatic ring), 1208 (C-O), and 1073 cm^{-1} (C-O-C).

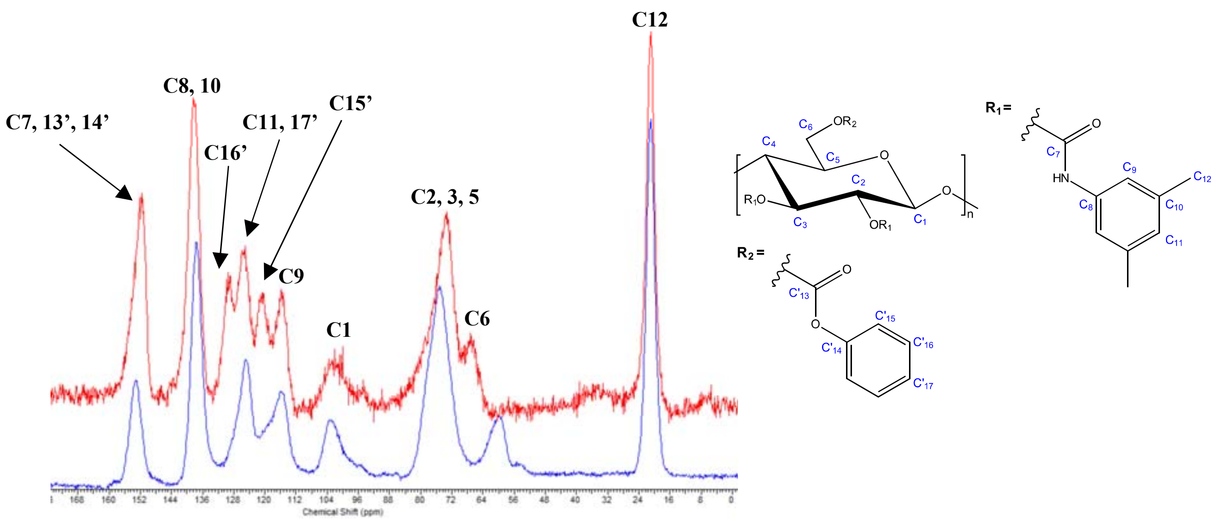

Fig. S5. Solid-state ^{13}C NMR spectra of cellulose derivatives **11c** (red) *vs.* **10** (blue), Cellulose derivative **11c**: δ = 102.1 (C1), 73.2 (C2, 3, 5), 67.2 (C6), 151.6 (C7, 13’, 14’), 138.3 (C8, 10), 115.8 (C9), 125.0 (11, 17’), 21 (C12), 120.7 (C15’), and 129.4 ppm (C16’).

The chemical formula of cellulose derivative **11c** was $C_{29.36}H_{29.21}O_{9.04}N_{1.84}$, the DS of phenyl carbonate was calculated by the formula:

$M_{\text{cellulose 6-}O\text{-(phenyl carbonate)-}bis\text{-2,3-}O\text{-(3,5-dimethylphenyl carbamate)}} =$

$M_{\text{cellulose 6-OH-}bis\text{-2,3-}O\text{-(3,5-dimethylphenyl carbamate)}} - M_H \times DS + DS \times C_7H_5O_2$

Where M is the molar mass.

Obtained: 24.96 g, yield = 98.8 %.

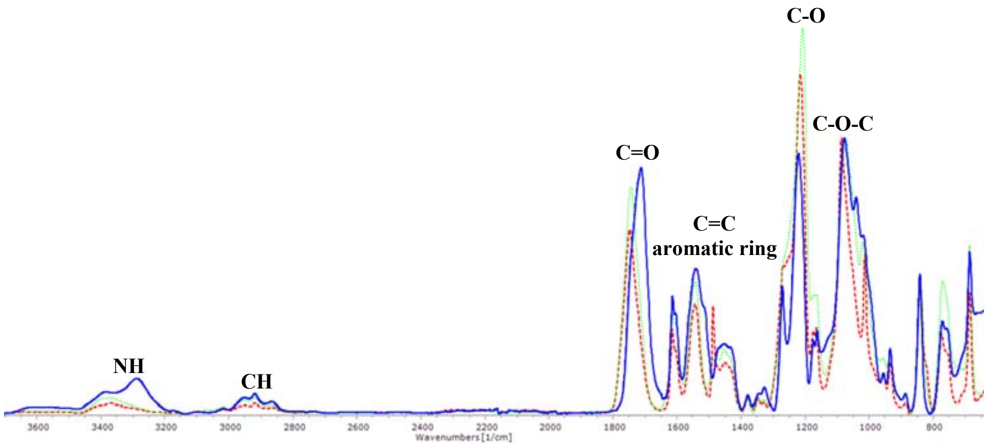

Fig. S6. FTIR spectrum of cellulose derivatives **12** (blue) *vs.* **11b** (dashed red) and cellulose derivative **11c** (dotted green),

Cellulose derivative **12:** υ = 3384, 3287 (NH), 2919 (CH), 1713 (C=O), 1614, 1543, 1453, 841 (C=C aromatic ring), 1221 (C-O), and 1078 cm^{-1} (C-O-C).

Solid-state ^{13}C NMR of cellulose derivative **12**: δ = 102.1 (C1), 73.1 (C2, 3, 5), 62.8 (C6), 152.8 (C7), 138.3 (C8, 10), 115.4 (C9), 124.8 (C11), 21.1 (C12), 155.3 (C13), 30.8 (C18), 88.7 (C19), and 81.0 ppm (C20).

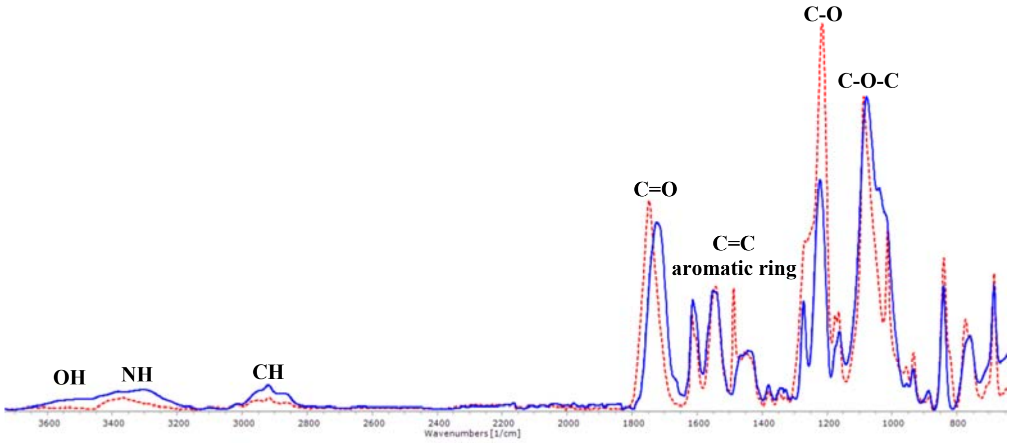

Fig. S7. FTIR spectra of cellulose derivatives **13aB** (blue) *vs.* **11b** (dashed red),

Cellulose derivative **13aB**: υ = 3529 (OH), 3303, 3287 (NH), 2920 (CH), 1721 (C=O), 1615, 1543, 1433, 841 (C=C aromatic ring), 1220 (C-O), and 1075 cm^{-1} (C-O-C).

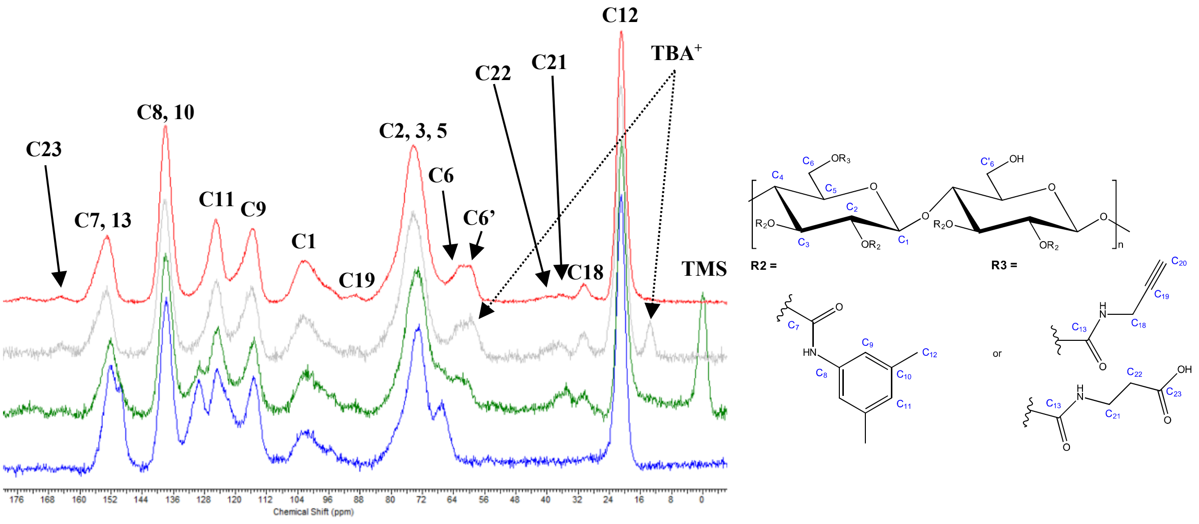

Fig. S8. Solid-state ^{13}C NMR spectra of cellulose derivatives **13aB** (green: crude product, grey: after purification with TBAF, red: after purification and protonation with HCl 0.1 M) *vs.* **11b** (blue),

Solid-state ^{13}C NMR of cellulose derivative **13aB**: 102.1 (C1), 74.4 (C2, 3, 5), 62.5 (C6), 59.9 (C6'), 153.0 (C7, 13), 138.0 (C8, 10), 115.7 (C9), 125.0 (C11), 21.0 (C12), 30.8 (C18), 88.9 (C19), 36 (C21), 39.7 (C22), and 174.7 ppm (C23).

Obtained: 2.32 g, yield: 83.9 %.

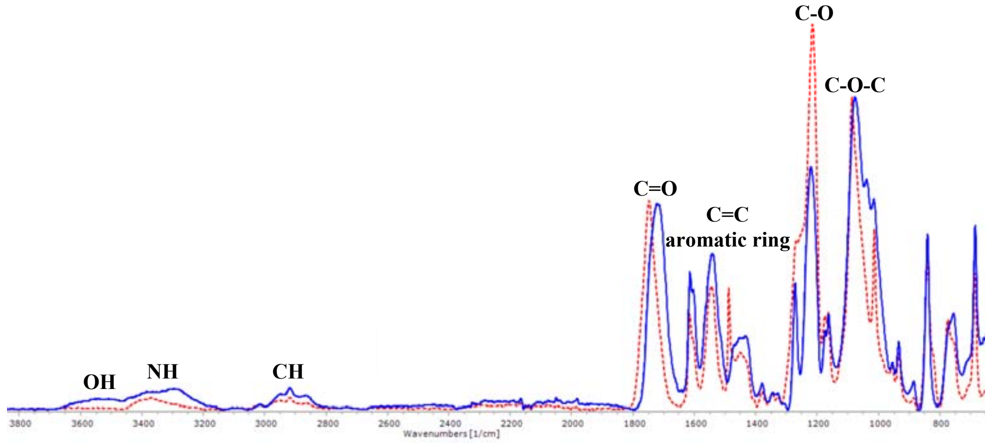

Fig. S9. FTIR spectra of cellulose derivatives **13bB** (blue) *vs.* **11b** (dashed red),

Cellulose derivative **13bB**: ʋ = 3529 (OH), 3296, 3287 (NH), 2918 (CH), 1722 (C=O), 1614, 1543, 1434, 840 (C=C aromatic ring), 1220 (C-O), and 1077 cm^{-1} (C-O-C).

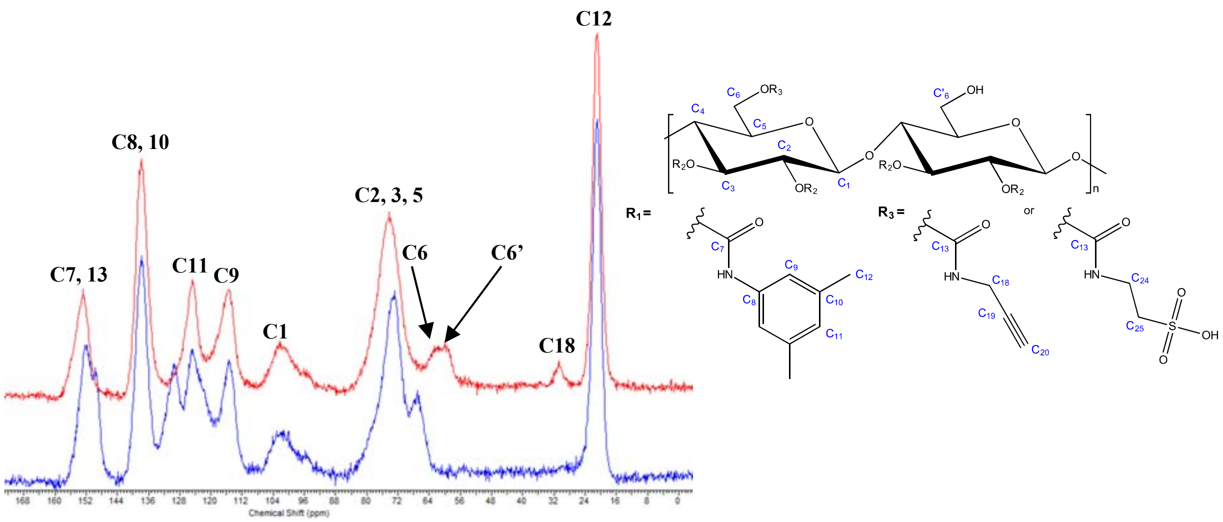

Fig. S10. Solid-state ^{13}C NMR spectra of cellulose derivatives **13bB** (red) *vs.* **11b** (blue), Cellulose derivative **13bB**: δ = 102.4 (C1), 74.2 (C2, 3, 5), 61.6 (C6), 59.9 (C6'), 152.8 (C7, 13), 137.9 (C8, 10), 115.4 (C9), 124.8 (C11), 20.8 (C12), and 30.8 ppm (C18).

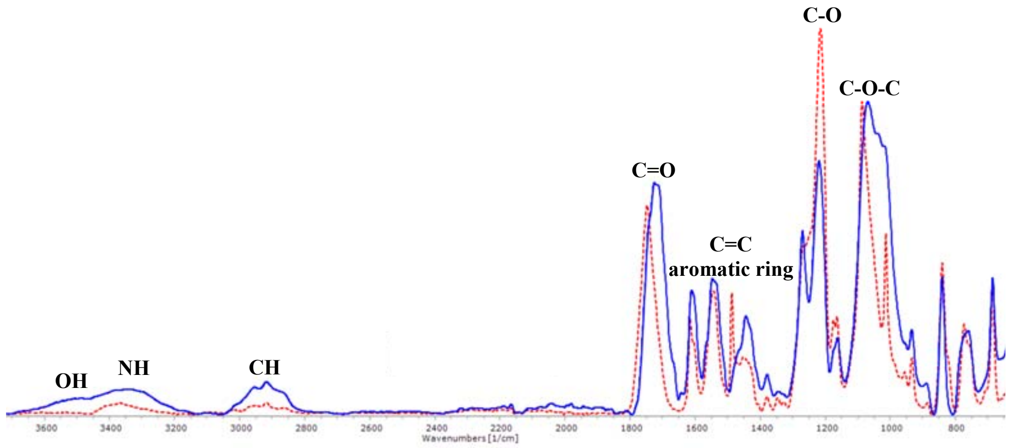

Fig. S11. FTIR spectra of cellulose derivatives **13aC** (blue) *vs.* **11b** (dashed red), Cellulose derivative **13aC**: υ = 3520 (OH), 3339 (NH), 2918 (CH), 1725 (C=O), 1610, 1548, 1444, 840 (C=C aromatic ring), 1270 (unknown), 1219 (C-O), and 1070 cm^{-1} (C-O-C).

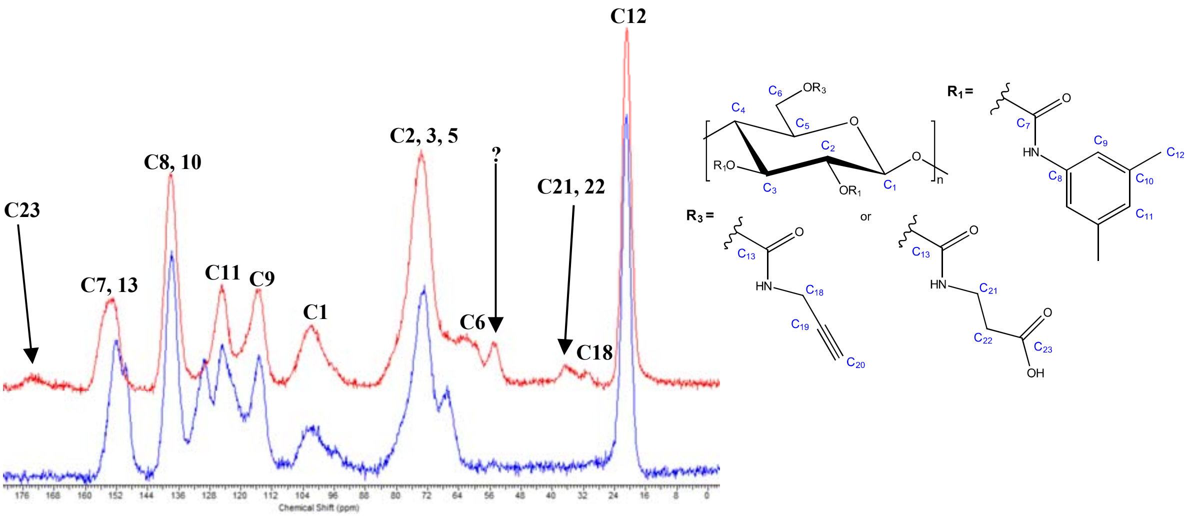

Fig. S12. Solid-state ^{13}C NMR spectra of cellulose derivative **13aC** (red) *vs.* **11b** (blue), Cellulose derivative **13aC**: δ = 101.7 (C1), 73.7 (C2, 3, 5), 62.0 (C6), 152.8 (C7, 13), 138.2 (C8, 10), 115.9 (C9), 124.8 (C11), 20.8 (C12), 31.0 (C18), 36.4 (C21, 22), 173.8 (C23), and 55.2 ppm (unknown).

Obtained: 2.22 g, yield: 76.8 %.

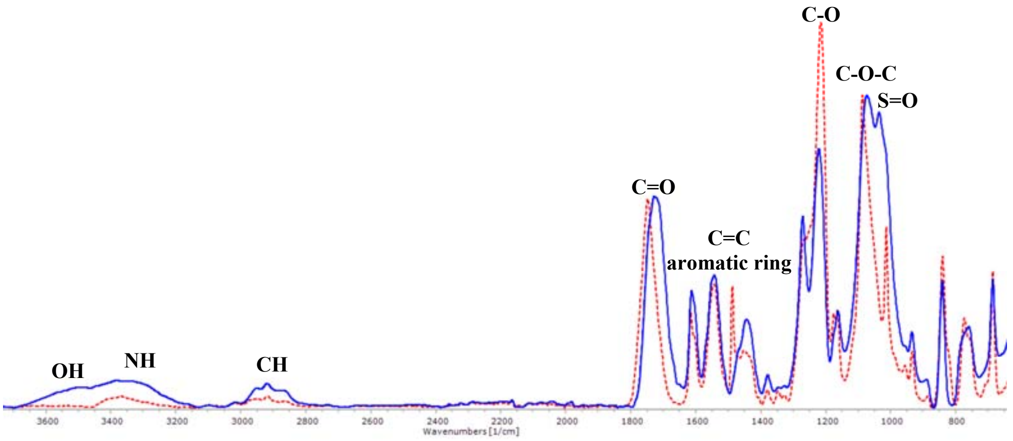

Fig. S13. FTIR spectra of cellulose derivatives **13bC** (blue) *vs.* **11b** (dashed red), Cellulose derivative **13bC**: υ = 3520 (OH), 3382 (NH), 2919 (CH), 1728 (C=O), 1613, 1543, 1444, 841 (C=C aromatic ring), 1270 (unknown), 1221 (C-O), 1073 (C-O-C), and 1035 cm^{-1} (S=O).

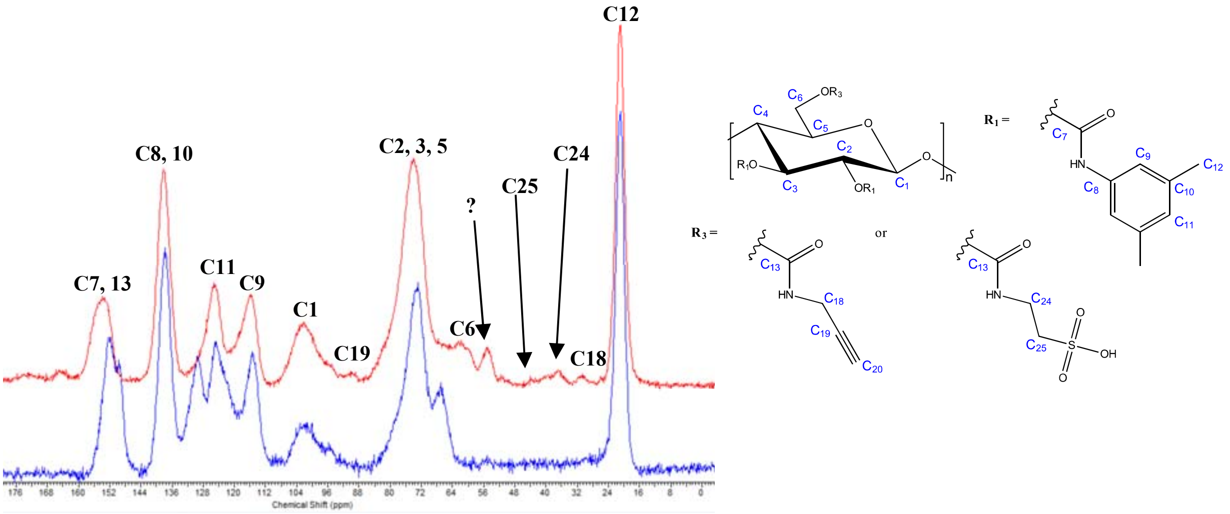

Fig. S14. Solid-state ^{13}C NMR spectra of cellulose derivatives **13bC** (red) *vs.* **11b** (blue), Cellulose derivative **13bC**: δ = 102.4 (C1), 74.1 (C2, 3, 5), 61.8 (C6), 153.8 (C7, 13), 138.2 (C8, 10), 115.8 (C9), 125.2 (C11), 21.0 (C12), 30.3 (C18), 89.8 (C19), 36.8 (C24), 50.9 (C25), and 55.2 ppm (unknown).

Obtained: 2.37 g, yield: 88.9 %.

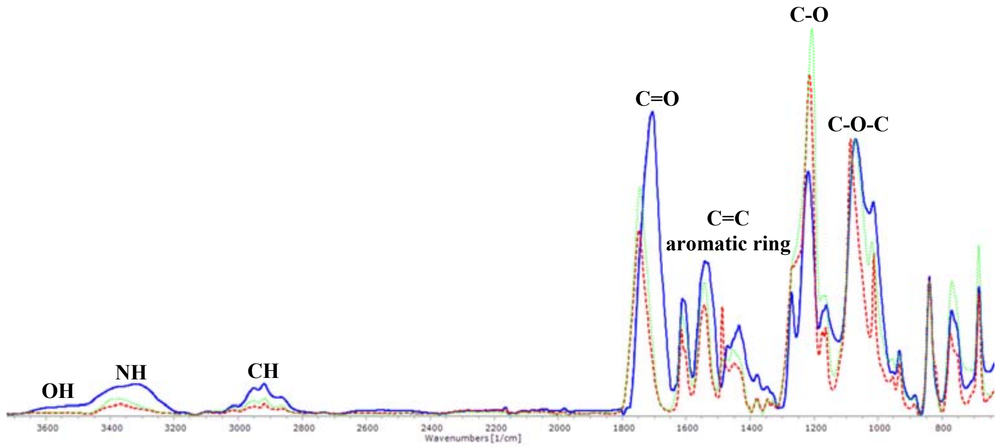

Fig. S15. FTIR spectra of cellulose derivative **13aD** (blue) *vs.* **11b** (dashed red) and **11c** (dotted green),

Cellulose derivative **13aD**: υ = 3520 (OH), 3378, 3313 (NH), 2919 (CH), 1708 (C=O), 1612, 1547, 1435, 840 (C=C aromatic ring), 1221 (C-O), and 1071 cm^{-1} (C-O-C).

Solid-state ^{13}C NMR of cellulose derivative **13aD**: δ = 103.4 (C1), 73.7 (C2, 3, 5), 62.5 (C6), 152.6 (C7), 138.2 (C8, 10), 115.7 (C9), 125.0 (C11), 21.0 (C12), 155.9 (C13), 36.8 (C21, 22), and 175.1 ppm (C23).

Obtained: 2.29 g, yield: 97.9 % with **11b** as a precursor.

Obtained: 2.37 g, yield: 95.6 % with **11c** as a precursor.

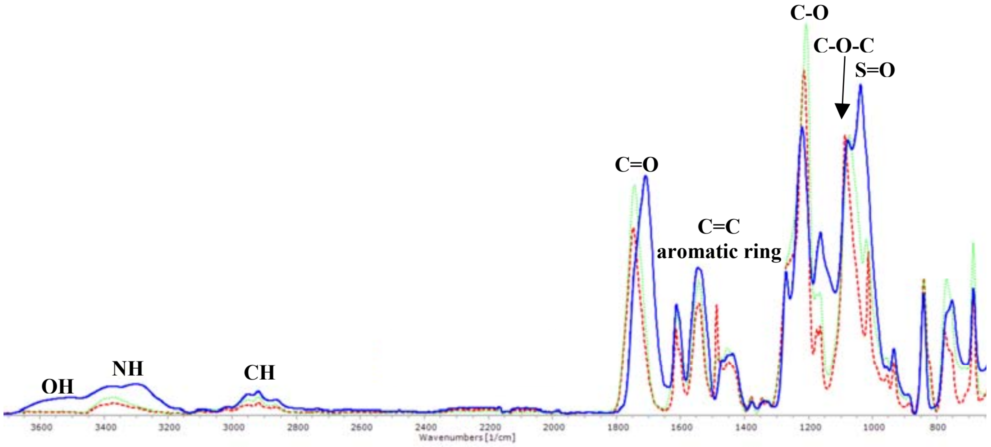

Fig. S16. FTIR spectra of cellulose derivative **13bD** (blue) *vs.* **11b** (dashed red) and **11c** (dotted green),

Cellulose derivative **13bD**: υ = 3520 (OH), 3378, 3302 (NH), 2919 (CH), 1708 (C=O), 1613, 1546, 1438, 841 (C=C aromatic ring), 1221 (C-O), 1078 (C-O-C), and 1038 cm^{-1} (S=O).

Solid-state ^{13}C NMR of cellulose derivative **13bD**: δ = 101.8 (C1), 73.7 (C2, 3, 5), 62.5 (C6), 153.3 (C7), 138.3 (C8, 10), 115.5 (C9), 125.2 (C11), 21.0 (C12), 156.7 (C13), 30.3 (C18), 37.0 (C24), 51.0 ppm (C25).

Obtained: 2.28 g, yield: 91.7 % with **11b** as a precursor

Obtained: 2.44 g, yield: 92.6 % with **11c** as a precursor.
